# Complete Genome Sequence of *Streptococcus iniae* UEL-Si1, Isolated in Diseased Nile Tilapia (*Oreochromis niloticus*) from Northern Paraná, Southern Brazil

**DOI:** 10.1128/genomeA.01458-16

**Published:** 2017-01-12

**Authors:** Laurival A. Vilas-Boas, Selwyn A. Headley, Kátia B. Gonçalves, Josiane A. Scarpassa, Lucienne G. Pretto-Giordano

**Affiliations:** a Department of General Biology, Universidade Estadual de Londrina, Londrina, Brazil; b Department of Veterinary Preventive Medicine, Universidade Estadual de Londrina, Londrina, Brazil

## Abstract

The *Streptococcus iniae* UEL-Si1 strain was isolated from diseased Nile tilapia within the Paranapanema River Basin, Northern Paraná, Brazil. This is an emerging infectious disease agent of fish from Brazil, and sequencing of the complete genome is fundamental to understanding aspects relative to pathogenesis, infection, epidemiology, and immunity.

## GENOME ANNOUNCEMENT

*Streptococcus iniae* is a Gram-positive bacterium that was first identified from the freshwater dolphin, *Inia geoffrensis*, in association with golf ball disease (1). The first description of *S. iniae* as a human pathogen occurred in 1996 in patients with septicemia and concomitant endocarditis, meningitis, and arthritis (2). *S. iniae* was then considered as a disease of humans and aquatic mammals (3), is currently a potential zoonotic disease (4, 5), and is a major pathogen of fish worldwide (4, 6). In Brazil, *S. iniae* was initially associated with meningoencephalitis and septicemia in Nile tilapia (*Oreochromis niloticus*) from farms located within the state of Paraná, Southern Brazil in 2012 (7). Recently, we have isolated and identified this pathogen in diseased Nile tilapia from Southern Brazil that were reared in net cages within the Paranapanema River Basin, Northern Paraná, and with clinical manifestations of exophthalmos, erratic swimming, and melanosis (8). Consequently, this pathogen is of great concern in Brazil due to the direct impacts on the local fishing industry, the growing public health concerns worldwide, and primarily since this infectious disease agent is not a common pathogen associated with fish disease in this country. Therefore, the complete sequencing of this organism is fundamental to understanding aspects related to pathogenesis, infection, epidemiology, and immunity.

The complete genome of *S. iniae* UEL-Si1 was sequenced using Illumina HiSeq/MiSeq, with the paired-end sequence strategy, which resulted in a total of 3,227,205 reads of high quality. The genome was assembled by *de novo* strategy with the software SPAdes version 3.9.0 (9). The assembly yielded 60 contigs and contains 2,395,193 bp, with a GC content of 36.3%. The RAST server program (10) proposed that this *S. iniae* genome contains 2,426 coding sequences from 329 subsystems. These include features associated with the metabolism for carbohydrates (*n* = 308); synthesis of amino acids and derivatives (*n* = 155); protein metabolism (*n* = 153); cell wall and capsule (*n* = 133); DNA metabolism (*n* = 114); RNA metabolism (*n* = 100); cofactors, vitamins, prosthetic groups, and pigments (*n* = 83); synthesis of fatty acids, lipids, and isoprenoids (*n* = 73); virulence, disease, and defense (*n* = 65); membrane transport (*n* = 51); phages, prophages, transposable elements, and plasmids (*n* = 46); stress response (*n* = 39); regulation and cell signaling (*n* = 38); phosphorus metabolism (*n* = 35); cell division and cell cycle (*n* = 32); iron acquisition and metabolism (*n* = 24); respiration (*n* = 15); potassium metabolism (*n* = 5); and motility and chemotaxis (*n* = 2).

### Accession number(s).

This complete genome shotgun project of *S. iniae* was deposited at DDBJ/ENA/GenBank under the accession number MNAC00000000. The version described in this paper is the first version, MNAC01000000.
